# THE ROLE OF DEMENTIA FRIENDS IN CREATING STRONGER NETWORKS WITHIN FAITH-BASED COMMUNITIES

**DOI:** 10.1093/geroni/igz038.1689

**Published:** 2019-11-08

**Authors:** Christy M Nishita, Heather Chun

**Affiliations:** 1 University of Hawaii, Center on Aging, Honolulu, Hawaii, United States; 2 National Asian Pacific Center on Aging, Seattle, Washington, United States

## Abstract

Faith-based communities are uniquely positioned to support members of their congregation with dementia and their caregivers. Aligned with their ministry and service work, congregations can identify individuals who need help and provide needed instrumental and emotional support. This poster will present preliminary findings from a pilot study with a Chinese, Mandarin-speaking church and a Samoan church in Honolulu, Hawaii. Using the Dementia Friends USA curriculum, which was translated into Mandarin and Samoan, the pilot study examined the potential for the Dementia Friends approach to mobilize a congregation and support caregivers. Findings indicate that there was great interest and need for information and dementia within the church community. In addition, a strong ministry and high proportions of older congregation members were keys to the success of the pilot. Finally, misperceptions of dementia within the Chinese and Mandarin communities were common and needed to be addressed in order for church members to fully embrace Dementia Friends principles (i.e., it is possible to have a good quality of life with dementia.) Future work will further explore faith-based community characteristics and other program components that lead to improved support for persons with dementia and their caregivers, as well as stronger dementia-friendly, faith based communities.

